# Omic characterisation of multi‐component defences against the necrotrophic pathogen *Pyrenophora tritici‐repentis* in wheat

**DOI:** 10.1111/plb.13746

**Published:** 2025-02-07

**Authors:** L. C. Ferreira, F. M. Santana, S. M. M. Scagliusi, M. Beckmann, L. A. J. Mur

**Affiliations:** ^1^ Department of Life Sciences Aberystwyth University Aberystwyth Wales UK; ^2^ University of Florida, Everglades Research and Education Center Belle Glade FL USA; ^3^ Laboratório de Fitopatologia Passo Fundo Rio Grande do Sul Brazil

**Keywords:** Auxin, cytoskeletal reorganisation, *Drechslera tritici‐repentis*, salicylic acid, tan spot, *Triticum aestivum*

## Abstract

- Tan Spot disease is caused by the necrotrophic pathogen *Pyrenophora tritici‐repentis* (*Ptr*) and poses a significant threat to global wheat production. Therefore, novel sources of resistance need to be identified, coupled with a fuller mechanistic understanding of host responses to *Ptr.*
- Herein, we characterise the interaction between a ToxA‐positive *Ptr* strain and parental wheat lines from a multiparent advanced generation intercross (MAGIC) population.
- Genotypes displaying moderate resistance (‘Robigus’) or susceptibility (‘Hereward’) to *Ptr* challenge were identified and characterised through histological, metabolomic, and transcriptomic approaches. Histological investigations indicated the prominence of papilla‐based defences in Robigus. Transcriptomic analyses could link this to the expression of barrier‐related genes *i.e.* actin polymerisation, callose deposition, vesicle trafficking, and cellulose synthesis. Inhibiting actin polymerisation with cytochalasin E increased lesion numbers but did not augment lesion growth, suggesting the deployment of other defence mechanisms. These may be influenced by auxin, as its exogenous application exacerbated symptom development. Transcriptomic and metabolomic analyses in Hereward following challenge with *Ptr* suggested shifts in primary metabolism, affecting glycolysis, the TCA cycle, and the γ‐aminobutyric acid (GABA) shunt. Activation of salicylic acid (SA)‐associated genes, including NPR1 and WRKY33, was specific to Hereward, and exogenous SA increased susceptibility to *Ptr* in both genotypes.
- This study suggests barrier defences could be effective against *Ptr* as well as a lack of susceptibility factors like SA or the appropriate processing of IAA. These findings offer potential avenues for enhancing wheat resistance to *Ptr*.

## Graphical abstract

Resistance against Tan spot (caused by *Pyrenophora tritici‐repentis*) in wheat can involve cell wall defences or an absence of susceptibility factors.

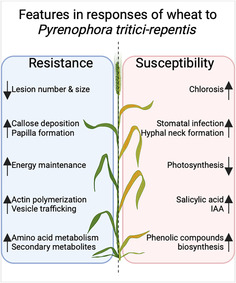

## INTRODUCTION

Tan Spot (TS) disease of wheat, caused by the fungus *Pyrenophora tritici‐repentis* (anamorph *Drechslera tritici–repentis*, Died) (*Ptr*) poses a significant threat to wheat crops. *Ptr* has the potential to cause yield losses up to 59% (Bhathal *et al*. 2003; Colson *et al*. 2003). The impact of TS is growing primarily due to the increasing fungicide resistance in *Ptr* populations (Sautua & Carmona 2021; Hoffmeister *et al*. 2022). Moreover, the pathogen's high plasticity, as revealed by the presence of giant transposons in its pan‐genome, is likely to drive the evolution of virulence in *Ptr* populations (Gourlie *et al*. 2022). Breeding superior varieties is a proven strategy in mitigating the risk of yield losses and fungicide resistance but also addressing the need for sustainable disease management (Buerstmayr *et al*. 2022). Germplasm exhibiting varying levels of resistance against TS has been identified (Phuke *et al*. 2020; Kokhmetova *et al*. 2021), but the new resistant varieties may need to be tailored to specific wheat‐growing regions. This is especially relevant in areas where TS is emergent, such as in the United Kingdom.

The plant immune system consists of a multifaceted defence response whose which components are exploitable for breeding. It encompasses structural barrier defences that limit pathogen intrusion and the production of enzymes and toxins to neutralise invading pathogens (Qalavand *et al*. 2023). These defence mechanisms may be either pre‐existing or inducible (Meisrimler *et al*. 2021). Induced defences are triggered by a series of pathogen recognition events, including “pathogen‐associated molecular‐pattern” (PAMP)‐triggered immunity (PTI). Pathogens, however, may evolve “effectors” which they deliver into the plant cytoplasm to suppress PTI and promote their own survival within the host. Effectors may in turn be recognised by the host resistance (*R*) genes, leading to effector‐triggered immunity (ETI), characterised by the hypersensitive response (HR), a form of programmed cell death. Disease often results from the absence of *Avr*/*R* gene product interactions (Flor 1971; Jones & Dangl 2006), with pathogens producing toxins, enzymes, and host‐modifying proteins (Westrick *et al*. 2021). Pathogens can be crudely classified as biotrophs (interacting with living host tissue) or necrotrophs (host cell death is a major symptom) or as a combination of both strategies—hemibiotrophs (Rajarammohan 2021). Discrete defence responses are required to be effective against biotrophs or necrotrophs mediated by different phytohormone signalling events. Most simply, salicylic acid (SA) mediated defences against biotrophs while jasmonates (JA) and ethylene (Et) are effective against necrotrophs (Monte 2023). Crucially, these two signalling modules can exhibit antagonistic interactions that some pathogens exploit to enhance disease progression (Kim *et al*. 2022).

*Ptr*, as a necrotrophic pathogen, follows an inverse gene‐for‐gene model in wheat, where necrotrophic effectors (NEs) produced by *Ptr* are recognised by susceptibility genes in the host to promote disease development (Wolpert *et al*. 2002; Strelkov & Lamari 2003). In the *Ptr*‐wheat pathosystem, three NE‐(*Tox* gene) host (*Tsc* gene) interactions have been described for, namely *ToxA‐Tsn1*, *ToxB‐Tsc2* and *ToxC‐Tsc1* (Ciuffetti *et al*. 2010; Singh *et al*. 2010; Faris *et al*. 2013). Additionally, qualitative resistance recessive alleles, namely *tan spot resistance* (*tsr*)*2*, *tsr3*, *tsr4* and *tsr5*, have been identified (Singh & Hughes 2006; Tadesse *et al*. 2006; Singh *et al*. 2008a, 2008b). One of the most‐well studied *Ptr* effectors is ToxA, a 13.2 kDa protein that is internalised in host cells via the chloroplast‐localised ToxA‐binding protein 1 (ToxABP1; Tuori *et al*. 1995; Zhang *et al*. 1997; Manning *et al*. 2007). This interaction may mediate ToxA‐induced necrosis (Lu *et al*. 2014), which is a product of light‐dependent accumulation of reactive oxygen species (ROS; Manning *et al*. 2009). ToxA also physically interacts with PR‐1‐5 (Lu *et al*. 2014), a pathogenesis‐related (PR) protein involved in SA‐dependent responses (Durrant & Dong 2004).

In this study, we used the parental lines of a wheat MAGIC (*i.e*., Multiparent Advanced Generation Inter‐Cross) population, developed by Mackay *et al*. (2014) and Gardner *et al*. (2016), to explore new sources of resistance against *Ptr* and investigate disease development. Among the selected lines, Robigus (Rob) exhibited significant resistance to *Ptr*, while Hereward (Her) was among the most susceptible lines. Through macro‐ and microscopic surveys and transcriptional and metabolomic comparisons of Rob and Her, we aimed to elucidate defence and disease‐associated changes following *Ptr* challenge. Our findings suggested the involvement of barrier defences, facilitated by cell wall appositions, along with cell and cytoskeleton rearrangements in the resistance mechanisms of Rob. SA associated events could also be susceptibility factor in Her.

## MATERIAL AND METHODS

### Plant materials and growth conditions

The wheat genotypes Alchemy, Brompton, Claire, Hereward, Rialto, Robigus, Soissons, and Xi‐19 constitute the eight founder lines of a MAGIC population sourced from the National Institute of Agricultural Botany (NIAB; UK, Mackay *et al*. 2014; Gardner *et al*. 2016). Seeds were sown into commercial compost John Innes no.2 and mixed with sand (4:1) and incubated in environmentally controlled growth rooms (Polysec, R. J. Hicks Refrigeration, Aberystwyth, UK) at 21 °C under 16 h of light, and at 18 °C in the dark. Each treatments had three replicates that were randomly distributed within the growth room. The plants were inoculated with *Ptr* at either of two growth stages: GS13 (three‐leaf seedlings) and GS65 (flowering/anthesis).

### *P. Tritici‐repentis* inoculum production

*P. tritici‐repentis* strains BR13, BR154, BR29 (all *Tox A*) were obtained from naturally infected wheat fields in Brazil and are part of the EMBRAPA Trigo collection. Conidia were produced and harvested following the methods described by Lamari & Bernier (1989). One *Ptr* mycelial plug was transferred to the centre of Petri dishes with V8 media (agar = 15 g; CaCO_3_ = 3 g; V8‐Juice = 150 ml, dH_2_O = 850 ml) and incubated at 25 °C for 5 days under continuous darkness to prevent conidiophore formation. The mycelia were flattened using the bottom of a sterilised test tube and ultrapure water. The mycelial slurry was discarded, and the plates were incubated under direct light for the next 24 h at 25 °C. To produce conidia, the plates were then transferred for a final incubation period of 24 h at 15 °C in darkness. The conidia were harvested using a paint brush and a solution of ultrapure water with 0.5% (v/v) Tween 80. The inoculum final concentration was adjusted to spores × 10^−3^ × ml^−1^ = 3.0. The inoculum was sprayed uniformly onto the leaves until run‐off. The inoculated plants were incubated in a humidity chamber for 24 h. Ultrapure water with 0.5% (v/v) Tween 80 was sprayed on control plants (mock‐inoculated treatment) which were incubated separately from the *Ptr*‐inoculated plants.

### Host classifications based on lesion scores

The host reactions to *Ptr* were assessed at 72, 120, 168, 240 and 336 h post inoculation (hpi) using a lesion‐based score system (Lamari & Bernier 1989). Score “1” indicated small, dark brown to black spots without any surrounding chlorosis or tan necrosis (suggesting resistant); “2”, small, dark brown to black spots with very little chlorosis or tan necrosis (suggesting moderately resistant); “3”, small, dark brown to black spots completely surrounded by a distinct chlorotic or tan necrotic ring, lesions, generally not coalescing (suggesting moderately resistant to moderately susceptible); “4”, small, dark brown or black spots completely surrounded with chlorotic or tan necrotic zones; some of the lesions coalescing (suggesting moderately susceptible); and “5” was given to plants which symptoms were dark brown or black centres with most lesions consist of coalescing chlorotic or tan necrotic zones (suggesting susceptible). Mean scores from 336 hpi were subjected to Pearson chi‐square testing to determine the independence between the genotypes and the resistance levels (Bonferroni corrected *P*‐value <0.05). RGB images of leaves were processed using ImageJ (Fiji) version 1.53c (Schindelin *et al*. 2012) to indicate lesion numbers. Statistical analyses were performed in R, using Kruskal–Wallis test followed by Dunn's test to determine significant comparisons (Bonferroni corrected *P*‐value <0.05).

### Histopathological analysis

Whole leaves were harvested at 24 and 72 hpi and cut into 0.5 cm pieces and vacuum infiltrated with ice‐cold 2.5% glutaraldehyde in 0.1 M sodium cacodylate at pH 7.2. The fixed samples were stored at 4 °C until analysed. Leaves were autoclaved at 121 °C for 2 min in 5 ml 1 M KOH solution, followed by three rinses with deionised water (Hood & Shew 1996). The specimens were mounted onto glass slides, stained with Aniline blue (0.05% water soluble aniline blue dye, No. 12642 George T. Gurr Ltd. London, in 0.067 M K_2_HPO_4_ pH 9) solution and viewed using an Olympus BX51 microscope fitted with an Olympus U‐RFL‐T‐200 UV Lamp. The images were capture with a ToupTex Industrial Digital Camera UCMOS08000KPB TP608000B. The micrographs were visually scored for single cell death, hyphal neck‐collars, papilla, infected stomata, and stomata with callose.

### Metabolomic assessments

The second or third fully extended leaves were excised from three seedlings from Robigus and Hereward at 0, 24, 48, 72 and 96 hpi. Each leaf was divided into two samples, each weighing approximately 40 mg (± 1 mg), totalling six biological replicates per treatment. These leaf samples were then placed in 2 ml sterile microcentrifuge tubes, each containing a 5 mm diameter acetone‐cleaned stainless‐steel bead. The samples were flash‐frozen in liquid N_2_ and homogenised using a ball mill system after which 1 ml of chloroform: methanol: dH_2_O (1: 2.5: 1) was added to each sample, followed by incubation in a shaker at 4 °C for 15 min. The samples were centrifuged at 5000 × g for 5 min and the supernatant was carefully transferred to a new microcentrifuge tube. From this, 100 μl of each sample was placed into a glass vial and sealed. Aliquots of 10 μl from each biological sample were pooled and used as a quality control (QC) sample. Untargeted metabolite fingerprinting was performed by Flow Infusion Electrospray Ionisation High‐Resolution Mass Spectrometry (FIE‐HRMS) based on a Exactive Orbitrap (Thermo Scientific, San Jose, CA, USA) mass spectrometer coupled to an Accela (Thermo Scientific) ultra‐performance liquid chromatography system. Pre‐mixed ultra‐pure H_2_O (18.2 Ω) and HPLC grade MeOH (Fisher Scientific) at a ratio of 7:3 or a flow solvent (mobile phase) were used to deliver 20 μl of the injected sample to the electrospray ionisation (ESI) source. For the first 1.5 min the flow rate was 200 μl·min^−1^ and 600 μl·min^−1^ for the subsequent 1.5 min. Both ionisation modes were acquired simultaneously. To acquire the mass spectra for each mode only one scan event was used with a scan rate of 1.0 Hz. The maximum injection time was 250 ms with a mass resolution of 100 000 and an automatic gain control (AGC) 5 × 10^5^. Mass‐to‐charge (*m/z*) analytes generated in negative and positive ionisation modes. Each sample was processed through the mass spectrometry twice, composing six additional technical replicates.

The peaks data was filtered based on relative standard deviation (RSD) of 0.5 and a minimum occupancy of 2/3 in each class. The *m/z* data was normalised based on total ion count (TIC) using the R package metabolyseR v0.14.10 (Finch 2022). Data are available in Table S5. Statistical analyses were performed in treatment/control pairwise comparisons of genotype and each time point; hereafter referred to as Rob_24, Rob_48, Rob_72, Rob_96, Her_24, Her_48, Her_72 and Her_96. Genotypic comparisons were performed on samples collected prior to inoculation (Rob_0/Her_0). Welch's Two Sample *t*‐test was used to identify differentially accumulating metabolites (DAMs), with adjusted *P*‐value <0.05.

Molecular formulas (MF) were assigned to significant features using the R package MFassign v0.7.7 (Finch 2023), using parameters based on the Seven Golden Rules which include LEWIS and SENIOR chemical rules as well as isotopic patterns (Kind & Fiehn 2007). However, in the absence of confirmation by tandem MS or Nuclear Magnetic Resonance (which would be logistically very difficult), the metabolites should be considered to be only tentatively identified. Functional analysis and pathway enrichment was performed by mapping the explanatory features to the KEGG metabolic network using the PageRank and Diffusion methods from the FELLA package (Picart‐Armada *et al*. 2018) and using *Aegilops tauschii* as the reference. Fold changes were calculated for *m/z* with assigned molecular formulas. In cases where a single MF were assigned to more than one *m/z* peak, the one with the highest intensity was selected. The peak intensity data for each *m/z* selected was summarised by mean in each subset of genotype/treatment/hpi after log_2_(x + 1) transformation. Fold changes were then calculated by taking the difference between inoculated (I) samples and the corresponding mock (M), and this difference was divided by the mock‐treated samples, *e.g*. (I‐M)/M. Fold changes for the genotypic comparison were calculated similarly, with Her_0 as the denominator.

### Transcriptomic analyses based on RNA‐seq

The second fully expanded leaf was harvested from three mock‐ and *Ptr*‐inoculated seedlings from Robigus and Hereward at three time points (0, 48 and 96 hpi). Total RNA was extracted using the RNeasy Plant Mini Kit (Qiagen, Hilden, Germany), with in‐column DNase digestion. RNA was quantified using a Nanodrop 1000 spectrophotometer (Thermo Scientific) and tested for RNA degradation and DNA contamination by agarose 1.2% gel electrophoresis. RNA integrity and quantitation assessments, library preparation, sequencing and raw data quality control were performed by Novogene (UK) Company Limited (Cambridge, UK). cDNA libraries were sequenced in an Illumina NovaSeq 6000, generating 150 bp‐long paired‐end reads.

Salmon version 1.3.0 (Patro *et al*. 2017) was used to map RNA sequencing reads to the reference transcriptome IWGSC RefSeq v1.1 (IWGSC (International Wheat Genome Sequencing Consortium) *et al*. 2018). The transcript counts were derived using the R package tximport 1.18.0 (Soneson *et al*. 2015). Differentially expressed genes (DEGs) with *P*‐value <0.05 and log_2_ fold change > ±1, identified with DESeq2, were functionally analysed by mapping against pathways categories using MapMan (Thimm *et al*. 2004; Love *et al*. 2014). Gene annotation was confirmed using the BLASTP program against *Aegilops*, *Oryza* and *Triticum* sequences. Counts were normalised using the variance stabilisation transformation (vst) function within DESeq2 package for data visualisation and clustering. Gene ontology annotations of the ref genome IWGSC was retrieved from (Ramírez‐González *et al*. 2018).

### Chemical treatments

Salicylic acid (SA), indole‐3‐acetic acid (IAA), and cytochalasin E (CytE) were obtained from Sigma‐Aldrich (Gillingham, UK). SA stock solution was prepared with absolute ethanol, whereas IAA was dissolved in 1 N NaOH, followed by dilution in dH_2_O to final concentrations of 500 and 10 μM. A cytochalasin E stock solution was prepared with 0.01% DMSO, then diluted to 10 μM in dH_2_O/0.2% (v/v) Silwet L‐77 (Newman Agrochemicals, London, UK) and sprayed onto plants 2 h prior to inoculation. IAA was sprayed to two separate groups of plants; with one group having application occur at 24 h before inoculation (designated IAA_pre) and with the other group 24 h post inoculation (IAA_pos). SA was only applied at 24 hpi.

### Lignin assessments

The lignocellulosic biomass of leaf samples was extracted and quantified using the method described by Barnes & Anderson (2017), with some minor modifications. Nine leaves were collected from each treatment at 0 and 96 hpi, immediately flash frozen in liquid N_2_ and freeze‐dried until moisture content reached ~10%. The specimens were placed in 2 ml microcentrifuge tubes containing a stainless‐steel ball and ground to fine powder in cryogenic mill. Pooled samples were subdivided into three replicates of 70 mg each. Biomass was fractioned to alcohol insoluble residue (AIR) by adding 1.5 ml of 70% ethanol, vortex and centrifuged for 10 min at 10,000 × *g*. The supernatant was removed, and the pellet was washed with 1.5 ml of chloroform: methanol (1:1 v/v), then with 1.5 ml of acetone to obtain the AIR. The samples were placed under a Reacti‐therm drying module at 35 °C until completely dried. The AIR mass was de‐starched by adding 1.5 ml of 90% DMSO (v/v) and shaken overnight in a platform rocker at 50 rpm. Samples were washed once in 90% DMSO, six times in 70% ethanol and once in acetone. The material was dried as described, and the absence of starch was verified by staining with Lugol solution (Sigma‐Aldrich). In between each extraction step, the samples were thoroughly vortex and centrifuged at 10,000 × *g* for 10 min. The derived lignocellulosic material was weighted and ~ 7 mg was used in acetyl bromide soluble lignin (ABSL) assay. Aliquots of 500 μl of 25% acetyl bromide (v/v) was added AIR samples (n = 3 replicates) in glass tubes and incubated at 55 °C for 3 h. Samples were vortexed every 10 min during the final hour. The reaction was terminated by adding of 5 ml of glacial acetic acid. AIR particles were allowed to settle overnight at room temperature. ABSL levels were measured at 280 nm in a spectrophotometer using a quartz cuvette with a solution containing 300 μl of the acetyl bromide/AIR solution mixed with 400 μl of 1.5 N NaOH and 300 μl of 0.5 M freshly made hydroxylamine hydrochloride. The reference reading was 400 μl of 1.5 N NaOH and 300 μl of 0.5 M freshly made hydroxylamine hydrochloride without the AIR samples. Acetyl bromide soluble lignin content was calculated using the Beer's Law, with an extinction coefficient of 19.81 g^−1^·L·cm^−1^. (Fukushima & Hatfield 2004). Statistical analyses were performed in R, using Tukey's HSD test to detect significant (Bonferroni corrected *P*‐value <0.05) interactions between “Genotype” and “Treatment”.

*In situ* lignin accumulation in *Ptr*‐infected tissues was visualised using phloroglucinol‐HCl (Ph‐HCl). The solution consisted of 3% phloroglucinol (Sigma‐Aldrich) in absolute ethanol mixed in 12 M HCl (1:1, v/v). Leaf segments were immersed in the Ph‐HCl solution and visualised with a Leica DM6000 B microscope mounted with Hitachi HV‐D20 3CCD camera.

## RESULTS

### Phenotypic characterisation of the parental MAGIC genotypes shows differential responses to *Ptr* infection

**Fig. 1:**
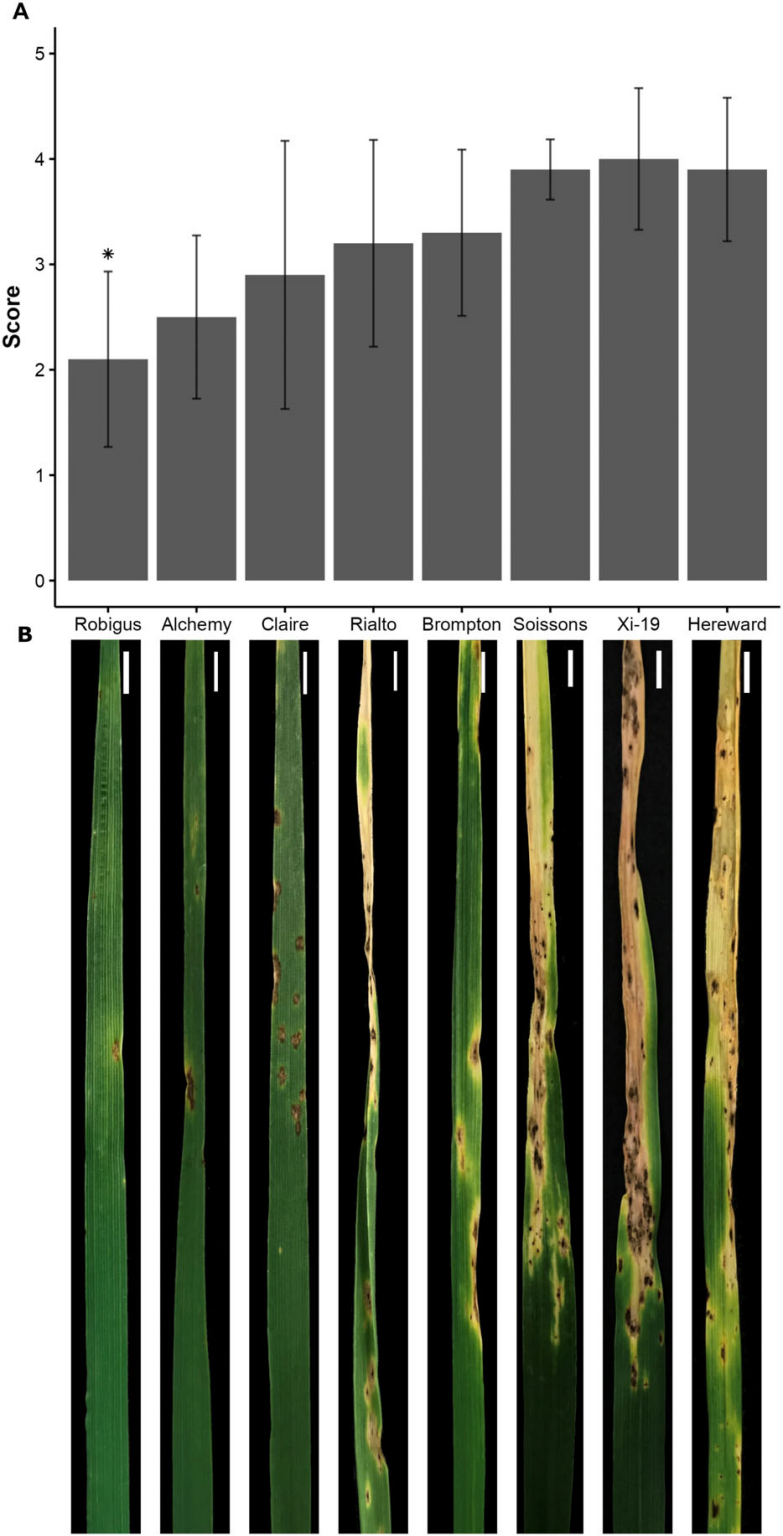
Phenotypic characterisation of MAGIC population founder lines regarding their reactions to Tan Spot (TS) disease. A: Mean scores (± SD) from seedlings and adult plants challenged with *Pyrenophora tririci‐repentis* strains BR13, BR29 and BR154. *P = 0.0039. B: Wheat leaves showing characteristic TS symptoms at 336 h post inoculation (hpi). Scale bar = 1 cm.

**Fig. 2:**
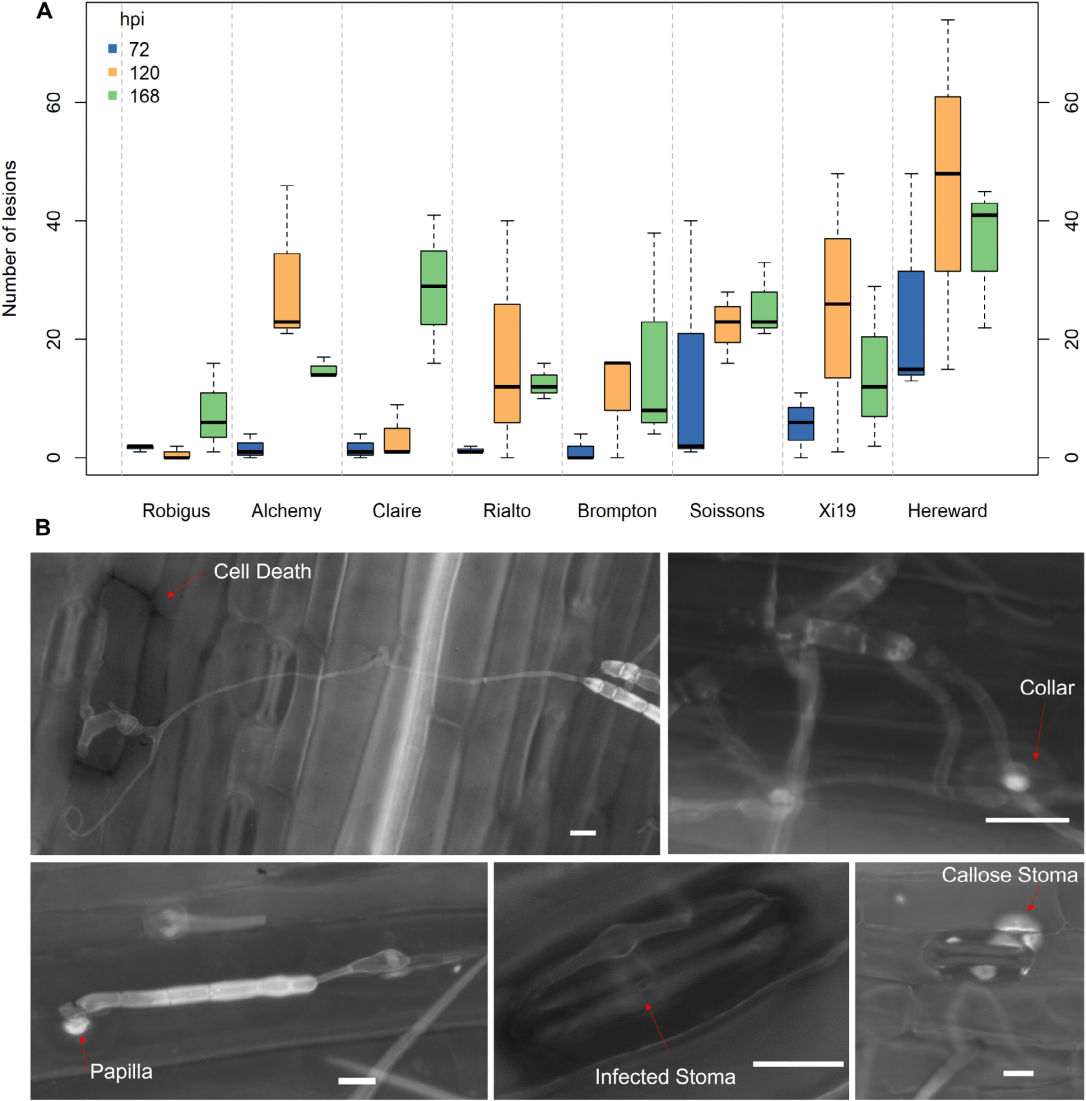
Reactions of wheat lines to *Pyrenophora tritrici‐repentis* infection. A: Number of lesions displayed in each wheat line at 72, 120 and 168 h post inoculation (hpi). B: Fluorescence microscopy showing categories of reactions observed, namely cell death, hyphal neck‐collar, papilla, infected stoma and callose stoma. Scale bar = 2 μm.

The eight parent genotypes of the wheat MAGIC population were screened for responses to *Ptr*. Adult plants (GS65 stage) and seedlings (GS13 stage) were challenged with strains of *Ptr* and scored for lesion development between 72 and 336 hpi (Table 1, Fig. 1). Average lesion scores at 336 h showed that Rob was the most resistant line and was classified as Moderately Resistant (MR). Alchemy, Claire, Brompton, and Rialto were classified as MR to Moderately Susceptible (MS) but Her, Soissons and Xi‐19 were MS (Fig. 1A). Classifications varied with pathogen strain and growth stage with plants being generally more susceptible to the most virulent strain (BR29) at the adult stage (Table 1). Symptom development in Rob was limited to a necrotic fleck with minimal chlorosis. Lesions in the most susceptible lines coalesced rapidly with extensive necrosis and chlorosis (Fig. 1B). Successful penetration events as indicated by macroscopic lesion numbers were significantly higher in MS genotypes than in Rob and MR‐MS genotypes (*P* = 0.0012 and 0.0069, respectively; Fig. 2A). The two most contrasting lines; Rob and Her, were selected for further characterisation.

**Table 1:** MAGIC population parental lines classification of Tan Spot resistance based on lesion types.

Growth stage/*Ptr* strain	Robigus	Alchemy	Claire	Rialto	Brompton	Soissons	Xi‐19	Hereward
Adults+Seedlings/BR13, BR29 and BR154	MR	MRMS	MRMS	MRMS	MRMS	MS	MS	MS
Seedlings/BR13, BR29 and BR154	MR	MRMS	MRMS	MRMS	MRMS	MS	MS	MS
Adults/BR29	MR	MS	MS	MRMS	MS	MS	MS	MS
Seedlings/BR13	MR	MR	MRMS	MS	MRMS	MS	MS	MS
Seedlings/BR29	MRMS	MRMS	MRMS	MS	MRMS	MS	MS	MS
Seedlings/BR154	MR	MR	MRMS	MRMS	MRMS	MS	MS	MS

MR (blue): moderately resistant; MRMS (orange): moderately resistant to moderately susceptible; MS (red): moderately susceptible.

Microscopic assessments of *Ptr* interactions with Rob and Her, indicated distinctive features occurring early following challenge; specifically, single‐cell death, papilla formation, callose deposition at stomata (Fig. 2B). Quantifying these different types of interactions, ~ 25% of infection sites in Rob at 24 hpi exhibited papilla formation, increasing to 43.9% at 72 hpi, respectively (Figure S1). Rob also showed greater callose deposition at stomata, which could contribute to the lower rates of infected stomata compared to Her. Her seemed to exhibit a preference for stomata infection (found in 60% of infection sites at 24 hpi) and hyphal neck formation which also can aid infection (Figure S1; Zhang *et al*. 2005).

### Transcriptomic assessments of the impact of *Ptr* inoculations of Robigus and Hereward

**Fig. 3:**
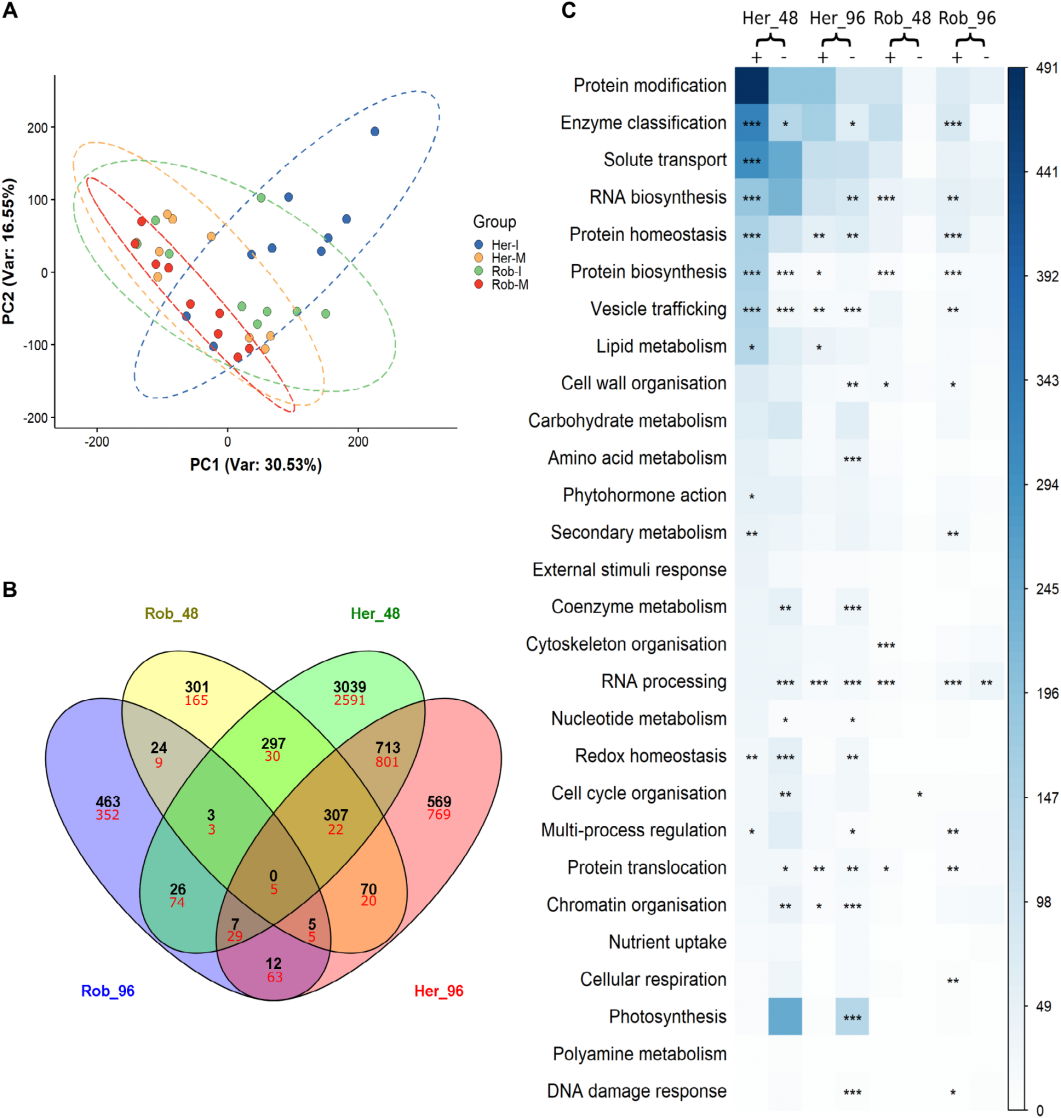
Analyses of transcriptomics data. A: Principal component analysis (PCA) displaying variations among biological replicates in mock‐treated (M) *versus* inoculated (I) samples from the wheat lines Robigus (Rob) and Hereward (Her). B: Venn diagram showing the number of differentially expressed genes (DEGs; *P* <0.05, log_2_ fold change >1) being up‐ (black) and down‐regulated (red) in Rob and Her at 48 and 96 h post inoculation (hpi). C: Distribution of DEGs from Rob and Her, at 48 and 96 hpi, in MapMan bins. Circle sizes and colour intensity represent the number of upregulated (+) and downregulated (−) DEGs mapped in each category. *P<0.05, ** *P*<0.01, ***P<0.001.

To understand the molecular mechanisms underlying resistance and susceptibility in wheat challenged with *Ptr*, we performed transcriptomic analyses. Leaves from the genotypes Rob and Her inoculated with the *Ptr* strain BR29, were sampled prior to visible symptoms at 0 and 48 hpi, and after symptoms were observed (96 hpi). RNA sequencing (RNA‐seq) generated 199,123,8260 reads, with an effectiveness (clean reads/raw reads) range of 97.14 to 99.37% (Table S1). Principal component analysis (PCA) showed that *Ptr* challenged Rob transcriptomes did not greatly change, with variation across PC1 (describing the major source of variation) was mostly explained by responses of Her to infection (Fig. 3A).

Transcripts (≥ 100 counts in three samples) were aligned to the IWGSC reference genome and linked to 42,276 different entries. Assessments of differentially expressed genes (DEGs) in Rob and Her before infection (*i.e*., Rob_0/Her_0) identified 2583 genes. Comparing all inoculated *versus* mock‐treated samples identified 15,193 DEGs. Focusing on 48 hpi, 1007 DEGs were upregulated and 259 were downregulated in Rob. However, wider‐ranging transcriptional changes were seen with Her at 48 hpi, with 4,392 genes being upregulated and 3,555 down‐regulated compared to controls (Fig. 3B). By 96 hpi, 965 DEGs were upregulated and 540 were downregulated in Rob, *versus* 1683 and 1714 DEGs in Her. Only 12 and 63 DEGs were either up or downregulated across all time points and genotypes (Fig. 3B).

Functional analyses of DEGs were based on mappings to GO annotations and MapMan bins, which are listed in Table S2. Our functional analysis initially assessed factors known to mediate interactions between wheat and *Ptr*. In both Her and Rob transcriptomes, we identified the presence of two *ToxABP1* transcripts and one *Tsn1* transcript (TraesCS5B02G059000.1). However, *Ptr* infection did not induce significant changes in their expression in either genotype. The transcripts TraesCS5A02G183300.1 and TraesCS7D02G161200.1, which are highly homologous to PR‐1‐5 (accession number = HQ541965; coverage = 100%; identity = 93.90% and 94.51%, respectively), were overexpressed in both lines challenged with *Ptr*.

Interrogating the DEGs for genes with known relevance to defence, transcripts coding for chitinases, wheatwin (PR‐4), phenylalanine ammonia lyase (PAL) were expressed in Rob prior to infection (Rob_0) and were further induced in all infected samples. A chitinase 8 transcript (TraesCS1D02G207000.1) and a PAL transcript (TraesCS6B02G258400.2) were 1.1 × 10^12^ and 1.04 × 10^6^ times (respectively) more highly expressed in Rob_0 compared to Her_0 (Table S2). Such pre‐existing factors with known roles in defence, may have contributed to enhanced *Ptr* resistance in Rob.

To investigate the biological processes differed between mock‐ and *Ptr*‐inoculated plants, DEGs were mapped on to MapMan pathways. The top five MapMan categories (representing 59% of DEGs in Rob_48 and 46% in Rob_96), were related to protein modification, enzyme classification, solute transport, RNA biosynthesis and protein homeostasis (Fig. 3C). Her was distinctive in showing 404 DEGs related to “photosynthesis” (Fig. 3C). Pathways linked to nucleotide metabolism, nutrient uptake, DNA damage response and redox homeostasis were poorly represented in Rob, with no more than 10 DEGs in each category. Changes in polyamine metabolism were not detected in Rob. The “not assigned” category was disregarded from this analysis.

**Fig. 4:**
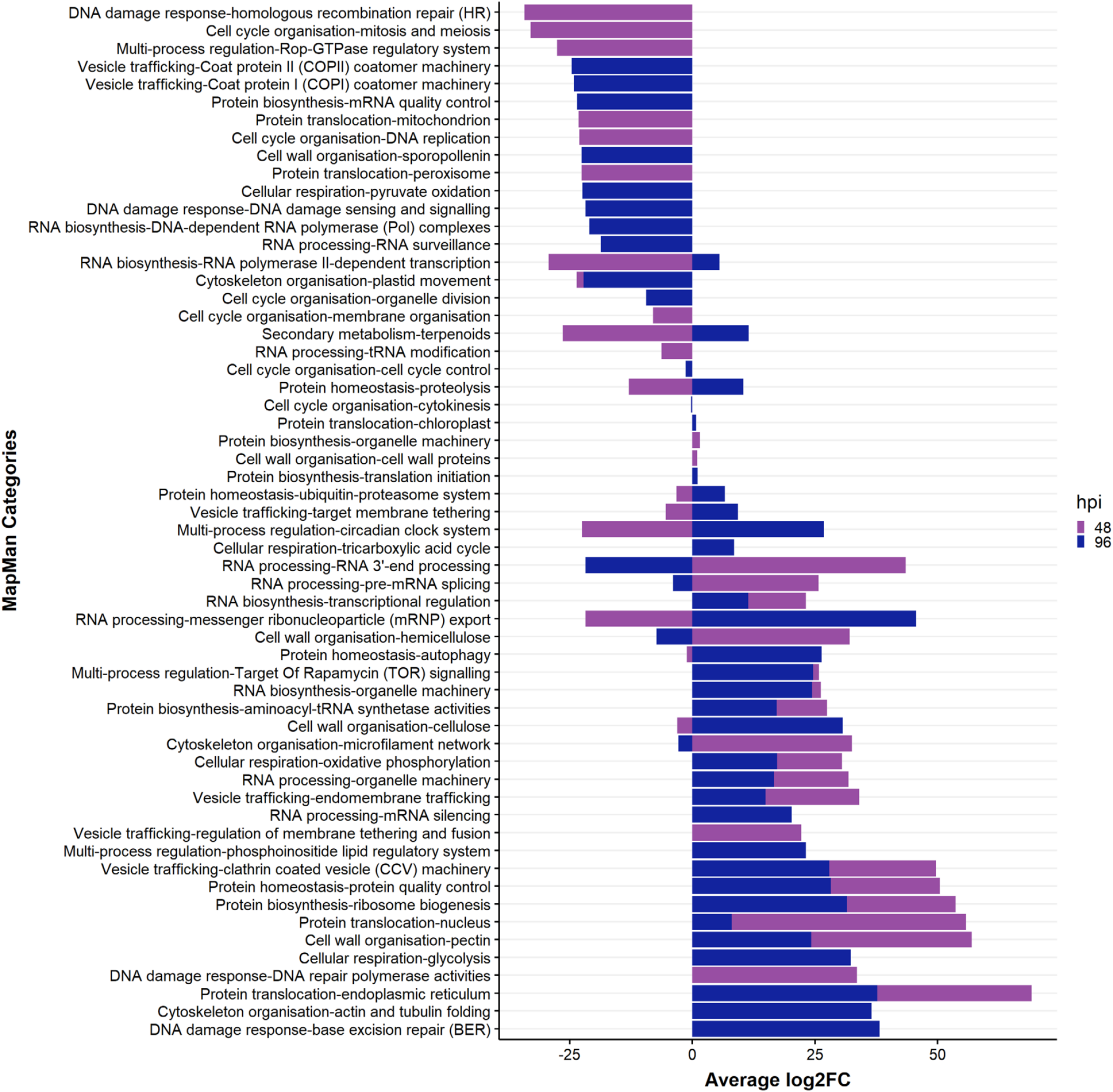
Significantly enriched MapMan bins found in the wheat line Robigus (Rob) in contrast to Hereward (Her) at 48 and 96 h post inoculation (hpi). The bars represent the average log_2_ fold change of transcripts mapped within first and second levels of the MapMan bins.

To identify key DEGs associated with resistance, we targeted MapMan bins that were present in Rob but were either absent or had opposite changes in Her. This yielded 245 unique bins to Rob (Fig. 4). The contrariwise comparison yielded 1303 bins unique to Her (Table S3). In Rob, we observed a substantial upregulation (average log_2_ fold change >15) of genes associated with vesicle trafficking, regulation of membrane tethering and fusion, clathrin‐coated vesicle (CCV) machinery, and endomembrane trafficking. Additionally, genes associated with cell wall and cytoskeleton organisation categories, including cellulose, hemicellulose, pectin, microfilament network, actin polymerisation and actin and tubulin folding, were also overexpressed. However, DEGs associated with organelle division, mitosis and meiosis, DNA replication and membrane organisation were repressed at either 48 or 96 hpi compared to Her. Considering cellular metabolism, genes involved in the Calvin cycle, oxidative pentose phosphate pathway (PPP), glycolysis and tricarboxylic acid (TCA) cycle, purines and pyrimidines and biosynthesis of amino acids were higher in Rob. Genes linked to photosystem I and respiration were significantly relatively higher in Rob_96 than Her.

In Her, the impairment of carbon assimilation and metabolism did not appear to result in the shift towards respiration, as genes involved in glycolysis and the TCA cycle were supressed (Table S3). Furthermore, GABA shunt related enzymes, including γ‐hydroxybutyrate dehydrogenase (GHB) and glyoxylate reductase (GR), were among the transcripts that were relatively low in Her. Considered together, defence in Rob is likely to be sufficient to protect against the metabolomic effects of TS lesion formation.

An important gene category differentially expressed in Rob compared to Her included components associated with phytohormones. Most striking was the relative over‐expression of BIG auxin transport gene in Rob (log_2_FC > 20) and their reduction in Her (log_2_FC = −22.881). Other auxin polar transport genes such as PINs were also downregulated in Her. However, Her did show auxin effects as also indicated with the up‐regulation of perception (TIR1/AFB) and efflux transporters (PILS) with infection (Table S3). Several components of the JA pathway were differentially expressed in Her, of which JAR1, AOC, JAZ, and OPC‐8:CoA synthetase were up‐regulated, whereas 13‐lipoxygenases were repressed. The upregulation of NPR1/3/4, salicylic acid 3‐hydroxylase and WRKY33 in Her could be associated with SA signalling and its effects (Figure S2).

### Metabolomic assessments of the impact of *Ptr* inoculations of Robigus and Hereward

**Fig. 5:**
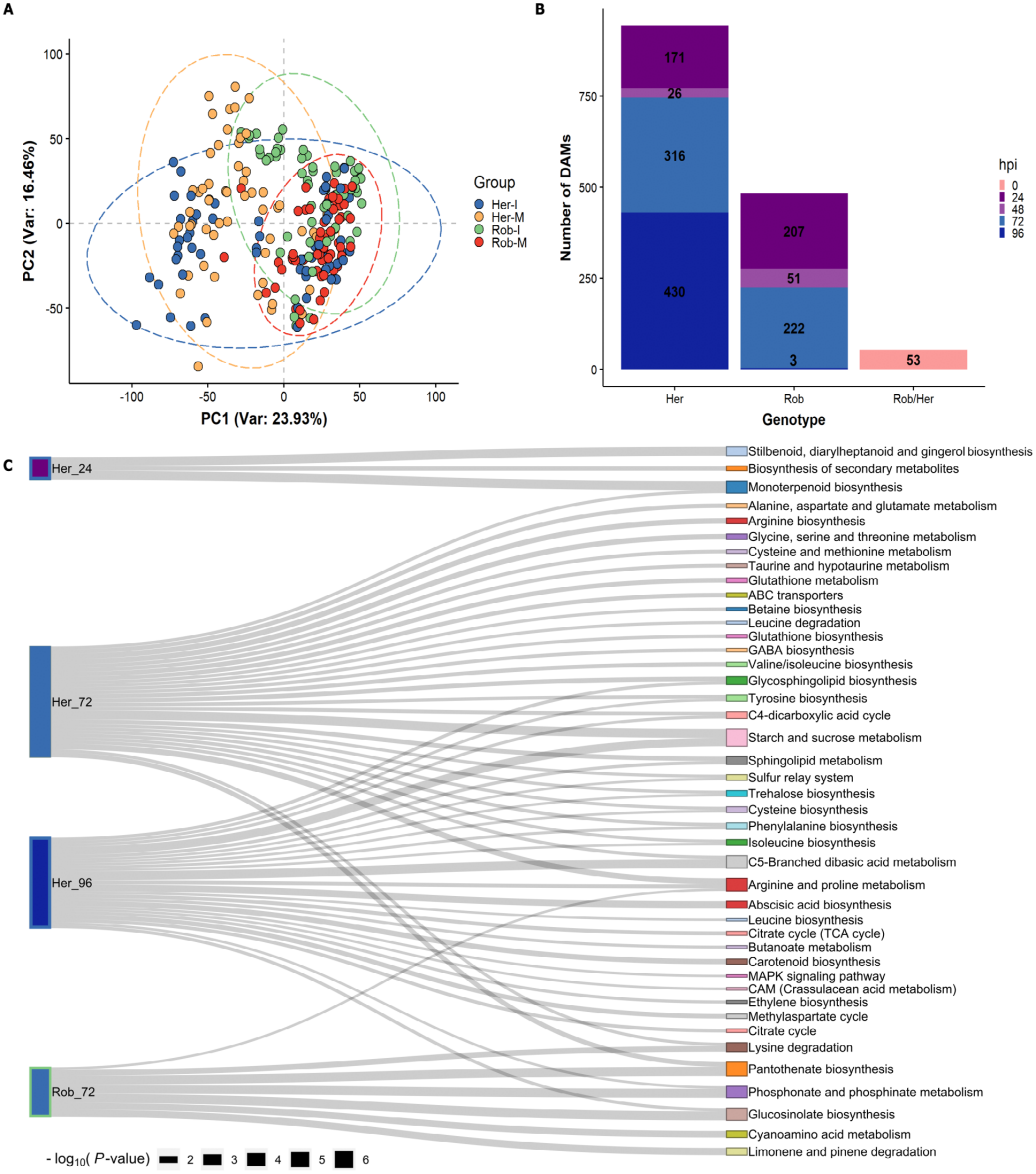
Statistical analyses on metabolomics data based on comparisons between mock‐treated and infected samples in the wheat lines Robigus (Rob) and Hereward (Her). A: Principal component analysis (PCA) displaying variations among biological replicates in mock‐treated (M) *versus* inoculated (I) samples from Rob and Her. B: Number of explanatory features (*P*<0.05) in each class, including the significant features detected in Rob in relation to Her before infection (0 hpi). C: KEGG pathway enrichment analysis of explanatory features from wheat lines Hereward (Her) and Robigus (Rob) at 24, 72 and 96 h post inoculation (hpi) with *Pyrenophora tritici‐repentis*. Time points not shown did not yield significant enrichments. The width of connector lines represents the enrichment significance (−log_10_(*P*‐value)) computed with the PageRank model, with wider connector lines indicating higher enrichment significance.

Our RNA‐seq analyses suggest that changes in the chemical composition of Robigus and Hereward could contribute to resistance and susceptibility to *Ptr*. To further explore this, we employed FIE‐HRMS to characterise the metabolomic profiles Rob and Her following challenge with *Ptr*. PCA showed that genotypic difference was the major source of variation across PC1 (Fig. 5A). We identified differentially accumulating metabolites (DAMs) by conduction pairwise comparisons between inoculated samples and their respective controls, as well as comparisons between genotypes before infection (Fig. 5B; Table S4). Tentative identifications were assigned to DAM when the MFs (with varied ionisations and isotopes) matched entries in KEGG. To understand the potential role of these DAMs in disease response, they were mapped to pathways in the KEGG database. Pre‐inoculation differences between the genotypes (53 DAMs), included the flavonoid quercetin 3‐O‐[beta‐D‐xylosyl‐(1‐ > 2)‐beta‐D‐glucoside] and the alkaloid, piperideine. As with transcriptomic profiling, *Ptr* infection triggered fewer changes in the Rob metabolome, compared to those in Her compared to controls. A total of 483 DAMs were detected between controls and infected samples in Rob, but there were twice as many DAMs in Her (Fig. 5B; Table S4). In Rob, most DAM changes were observed early in after inoculation, whilst in Her, they were at later timepoints. A significant increase in 13‐hydroperoxyoctadeca‐9,11,15‐trienoic acid (13(S)‐HPOT) which is linked to jasmonate biosynthesis, 3‐butyn‐1‐al, (S)‐malate and sucrose was seen only in Her. However, fatty acids, saponins, glycosides and amino acids, including γ‐aminobutyric acid (GABA) and L‐glutamate, were lower in Her following *Ptr* inoculation.

Analysing the pathways significantly enriched by DAMs could give us insights into the metabolic defences against *Ptr*. Enrichment analysis of KEGG pathways could target few changes in Rob prior to 72 hpi (Fig. 5C, Table S4). This was mainly due to the small number of DAMs in each time point, with no significant enrichment observed in Rob at 24, 48 and 96 hpi. However, DAMs from Rob_72 were associated with arginine and proline, phosphonate and phosphinate, cyanoamino acid metabolism, limonene and pinene degradation, glucosinolate biosynthesis, lysine degradation and pantothenate biosynthesis. For Her, stilbenoid, diarylheptanoid and gingerol, and monoterpenoid biosynthesis were over‐represented at 24 hpi (Fig. 5C). Her_72 and 96 shared 12 pathways, including phenylalanine and tyrosine biosynthesis. No significantly enriched pathways were observed in Her at 48 hpi.

### Validating features suggested as important components of responses to *Ptr*

**Fig. 6:**
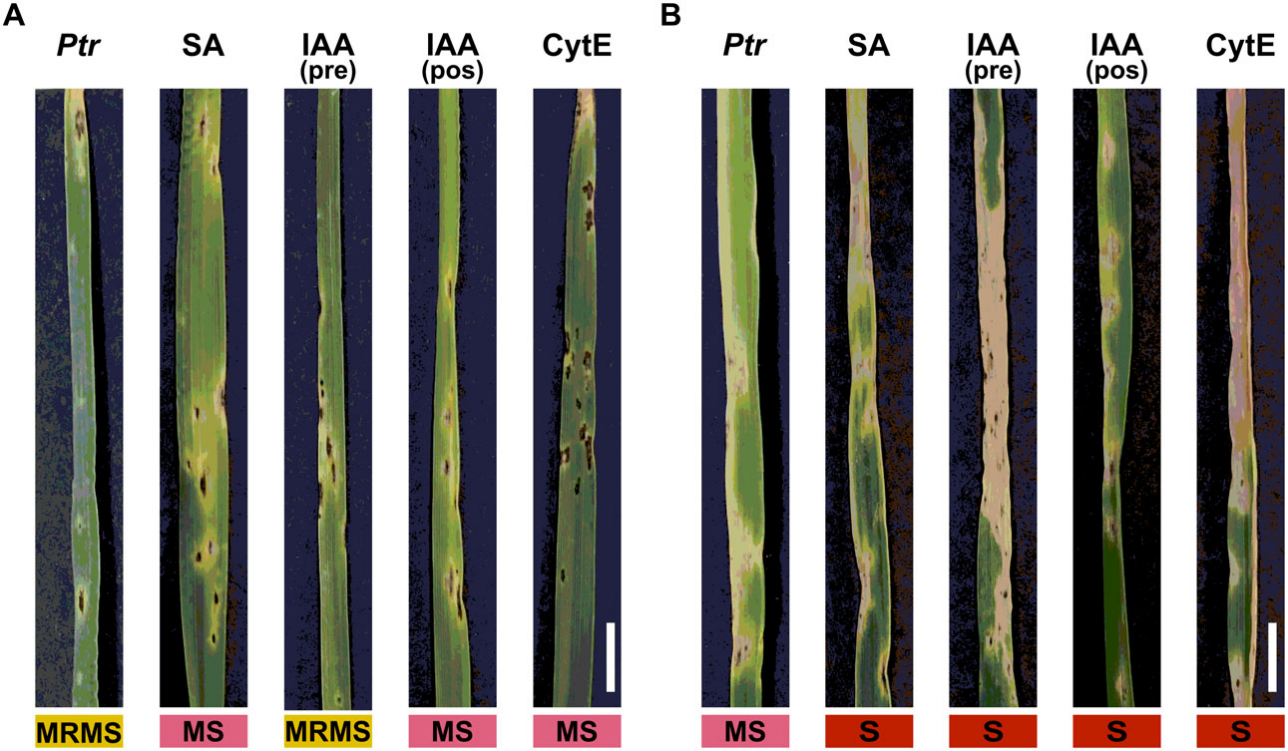
Phenotypic characterisation of seedlings of the wheat lines A: Rob and B: Her that were inoculated with *Pyrenophora tritici‐repentis* (*Ptr*) [positive control] and treated with salicylic acid (SA), auxin (IAA), and Cytochalasin E (CytE). Representative leaves displaying characteristic tan spot symptoms used for lesion‐based classification of resistance. The coloured boxes show the resulting host classification based on mean scores given at 336 h post inoculation with the *Ptr* strain BR29. MRMS, moderately resistant to moderately susceptible; MS, moderately susceptible; S, susceptible. Scale bar = 1 cm.

The combined analysis of our phenotypic and omic experiments suggests the importance of low fitness cost pre‐existing and inducible defences against *Ptr*, characterised by papilla formation and the activation of various genes/pathways associated with cell wall modifications. To validate these findings, we investigated the importance of cytoskeletal changes seen in Rob post *Ptr* infection. Seedlings from both genotypes were sprayed with cytochalasin E (CytE), an actin polymerisation inhibitor which effectively disrupts papilla formation (Kobayashi *et al*. 1997). CytE treatment resulted in increased susceptibility in both genotypes, but in Rob, this was manifested as an increase in lesion numbers, while lesion sizes hardly increased compared to controls, with little chlorosis observed around necrotic spots (Fig. 6).

To verify the effects of some key phytohormones in defences against *Ptr*, seedlings of Rob and Her were sprayed with SA and the auxin, indole acetic acid (IAA). JA was not examined as its effects are known to be antagonised by SA (El Oirdi *et al*. 2011) leading us to focus on the impact of SA. In Her, these treatments had no notable effect by at 336 hpi. However, in Rob treatment with either hormone increased chlorosis around dark lesions in Rob that reflect initial sites of infection (Fig. 6). This was consistent with IAA augmenting TS symptom development. Rob plants under all treatments displayed expanded chlorosis in comparison to untreated *Ptr*‐inoculated plants (Fig. 6).

Transcriptomic assessment had targeted PAL expression whilst metabolomic data has also indicated that phenylalanine and tyrosine biosynthesis was important in responses to *Ptr*. We sought to determine how far this could reflect changes in lignin contents as one output of PAL‐regulated phenylpropanoid metabolism with established defensive roles. Assessments indicated that Her had higher lignocellulosic content compared to Rob (Fig. S3A) although microscopic analyses of phloroglucinol stained leaves did not show lignification at the infection sites of either genotype (Figure S3). This suggested that effects were not linked to detectable changes in lignification following *Ptr* challenge.

## DISCUSSION

MAGIC populations were generated to increase recombination events and enhance mapping precision (Gardner *et al*. 2016), thereby providing resources that could be used in breeding such as disease resistance. To exploit the opportunities represented by the MAGIC population, we screened the parental genotypes for reactions to *Ptr* infection to identify new sources of TS resistance and to better understand wheat‐*Ptr* pathophysiology. Of the eight genotypes investigated, Rob was identified as the most resistant line (Fig. 1, Table 1). This is a widely cultivated UK line, that has been frequently used as a parent in breeding programmes (Fradgley *et al*. 2019) and also exhibits resistance to wheat midge and ergot (Gaafar *et al*. 2011; Gordon *et al*. 2015). We also identified three parental lines with significance TS susceptibility, namely Her, Xi19, and Soissons. These genotypes were also targeted by Corsi *et al*. (2020) as exhibiting sensitivity to ToxB. In our study, Her was chosen to represent a susceptible interaction and is a popular bread‐making UK variety, whose grain development and composition have been well‐characterised (Shewry *et al*. 2012; Min *et al*. 2020). By comparing phenotypic observations, we found a significant correlation between MR and MRMS classifications with lower number of infection sites and higher frequency of cell wall appositions (Fig. 2, Table 1). This suggests the importance of barrier defences against *Ptr* as in the wheat‐powdery mildew pathosystem (Bhuiyan *et al*. 2009; Bellincampi *et al*. 2014).

**Fig. 7:**
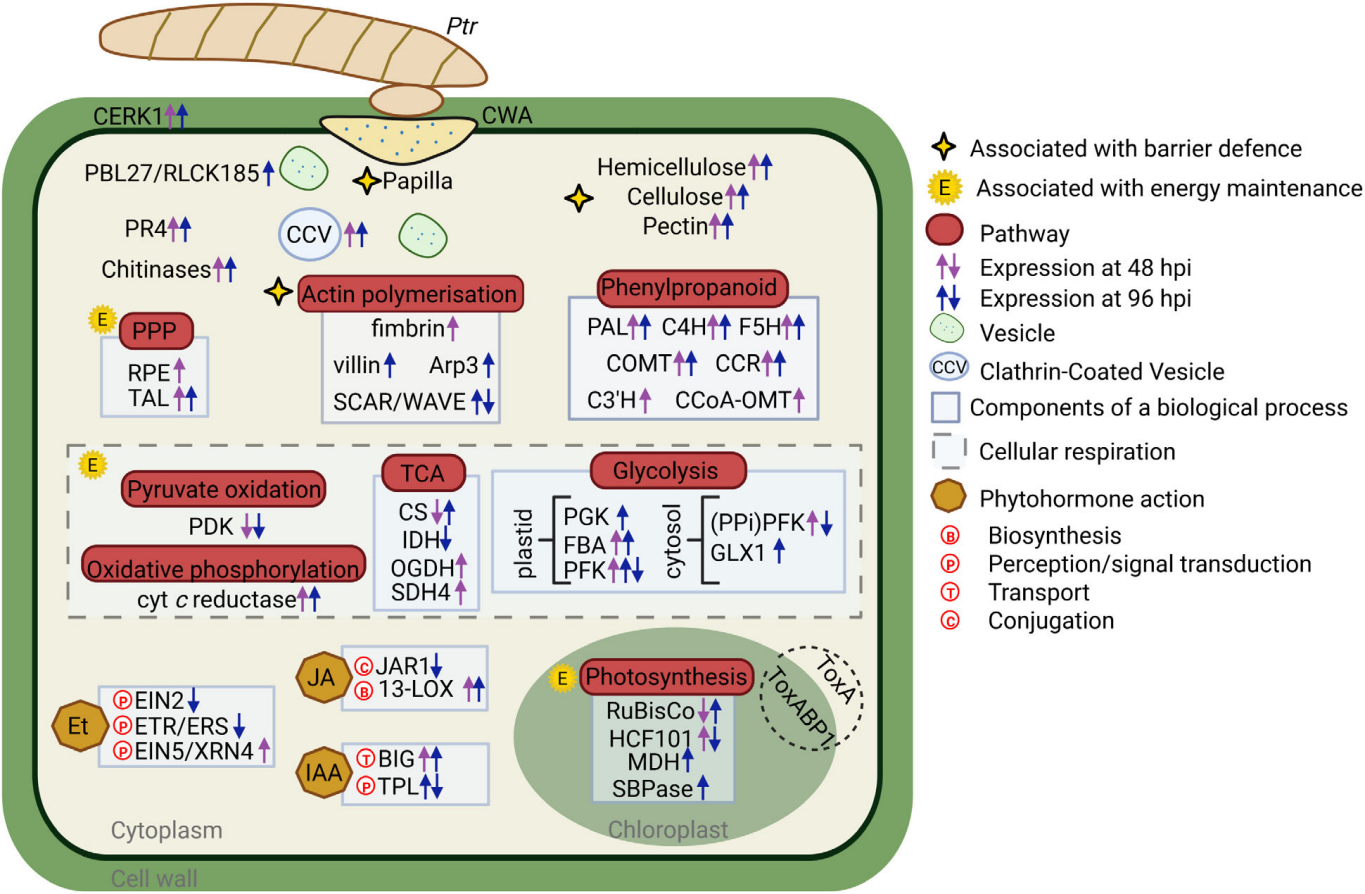
Schematic representation of events associated with moderate resistance of wheat line Robigus (Rob) to ToxA‐producing *Pyrenophora tritici‐repentis* (*Ptr*). Barrier defence and energy maintenance were highlighted as features of resistance. The barrier defence mechanism included papilla formation, cell wall appositions (CWA), overexpression of genes associated with hemicellulose, cellulose, pectin, Actin polymerisation and vesicle trafficking. The other resistance feature was characterised by the modest metabolomic response, the preservation of photosynthetic components, and by the maintenance of pentose phosphate pathway (PPP) and cellular respiration, as means of TCA cycle, glycolysis, and oxidative phosphorylation. Responses also included the activation of chitin‐induced defences, such as the chitin receptor CERK1 and its phosphorylation derivative PBL27/RLCK185, as well as chitinases and PR‐4 genes. Transcriptional changes underlying phytohormone action were also prominent within the first 96 h post inoculation (hpi), with emphasis on the overexpression of BIG auxin/IIA transporter. Abbreviation names can be found in the text or hereafter. (PPi)PFK, pyrophosphate‐dependent phosphofructokinase. 13‐LOX, 13‐lipoxygenase. C3′H, p‐coumaroyl shikimate/quinate 3′‐hydroxylase. C4H, cinnamate 4‐hydroxylase. CCR, cinnamoyl‐CoA reductase. CCoA‐OMT, caffeoyl‐CoA 3‐O‐methyltransferase. COMT, caffeic acid O‐methyltransferase. CS, citrate synthase. EIN2, EIN2‐type ethylene signal transducer. EIN5/XRN4, EBF‐modulating exoribonuclease. ETR/ERS, ETR/ERS‐type ethylene receptor protein. F5H, ferulate 5‐hydroxylase. FBA, fructose‐1,6‐bisphosphate aldolase. HCF101, Fe‐S cluster assembly factor involved in PS‐I assembly. GLX1, lactoyl‐glutathione lyase. IDH, isocitrate dehydrogenase. JAR1, jasmonoyl‐amino acid synthetase. MDH, NADPH‐dependent malate dehydrogenase. OGDH, 2‐oxoglutarate dehydrogenase. PFK, ATP‐dependent phosphofructokinase. PGK, phosphoglycerate kinase. RPE, ribulose‐phosphate 3‐epimerase. SDH4, membrane‐anchor component SDH4 of succinate dehydrogenase complex. SBPase, sedoheptulose‐1,7‐bisphosphatase. TAL, transaldolase. Created in biorender.com.

To further understand the molecular mechanisms associated with resistant and susceptible phenotypes, we undertook transcriptomic and metabolomic assessments of wheat‐*Ptr* interactions. It was notable that few changes occurred in the transcriptomic and metabolic profiles of infected leaves of Rob compared to controls (Figs 3 and 5). This was in contrast to the large scale transcriptional and metabolic reprogramming often reported during defence against infection (Hahlbrock *et al*. 2003; Mareya *et al*. 2019) and suggests that the form of resistance to *Ptr* was relatively subtle. This was consistent with most molecular responses occurring only in a few cells at, or adjacent to, infection sites. A schematic of the hypothesised events associated with moderate resistance to *Ptr* in Rob is shown in Fig. 7.

Our RNA‐seq data suggested the expression of *tsn1* in both Rob and Her indicating potential susceptibility to ToxA effects. In sensitive hosts, ToxA is internalised and is then localised to the chloroplasts leading to photosystem disruption with resulting ROS accumulation (Manning & Ciuffetti 2005; Manning *et al*. 2008). Transcriptome changes induced by isolated ToxA and ToxB treatments have been well‐characterised in susceptible plants (Pandelova *et al*. 2009, 2012). These studies showed the upregulation of v‐ and t‐SNARE, PAL, transcription factors (WRKY), JA and Et events; and the downregulation of PSI and PSII, translation and chloroplastic oxidative detoxification. These patterns aligned with the observed changes seen in Her suggesting a role for ToxA in this interaction (Table S2). It was not possible to define a role for ToxA in Rob but gene expression patterns indicated that photosynthesis was maintained (Figs 1B, 4). This is likely a simple effect of poorer fungal penetration of Rob but ToxA detoxification or even a protection of photosynthesis could be features of the resistance.

Examining differentially expressed genes suggested the induction of cell and cytoskeleton modifications as well as vesicle trafficking in Rob (Fig. 4). It is known that the actin cytoskeleton forms a network of microfilaments at infection sites, which, along with accumulation of secreted cell components can lead to penetration resistance (Takemoto *et al*. 2003; Hardham *et al*. 2007; Li & Day 2019), and therefore aligns with the microscopic assessments of responses to *Ptr* challenge in Rob (Fig. 2B). We provided further evidence of this through the application of CytE prior to challenge with *Ptr*. Cytochalasins are known to depolymerize the actin cytoskeleton and has been used widely to investigate cytoskeletal effects on plant resistance to pathogens (Moral *et al*. 2017; Li & Staiger 2018). Thus, treatment with CytE suppressed (*e.g*.) the resistance of *Arabidopsis thaliana* against *Pseudomonas syringae* and *Colletotrichum* spp. and barley against by *Blumeria graminis* f. sp. *hordei* (Miklis *et al*. 2007; Shimada *et al*. 2007; Kang *et al*. 2014). In line with this, we observed increased penetration of Rob with CytE treatments as indicated by increased lesion numbers (Fig. 6).

Auxin appears to play a complex role in the *Ptr‐*wheat interaction. Thus, we observed differential induction of BIG polar auxin transport in Rob, whereas the susceptible line downregulated several IAA transporter transcripts, such as PIN. Auxin transport via BIG have been demonstrated to enhance resistance against *Fusarium oxysporum* in Arabidopsis (Gil *et al*. 2001; Kazan & Manners 2009; Liu *et al*. 2015). Possibly, a key observation is that auxin suppress actin bundling and interfere with vesicle trafficking (Dhonukshe *et al*. 2008; Chang *et al*. 2015). This was suggested by the increased susceptibility that we observed with Rob with exogenous application of IAA (Fig. 6). Taking these observations together, it may be that in Rob, IAA transport could be occurring away from infection sites thereby favouring barrier defences.

The slower growth lesion rates seen in Rob led us to hypothesise additional defence mechanisms were deployed after penetration resistance. However, neither the transcriptional nor the metabolomic data provided a clear indication of an induced defence in Rob. Instead, it may be that Rob lacks susceptibility factors that are present in Her. In particular, SA associated events appeared to be being activated in Her. The antagonistic relationship between SA and JA in plant pathogen interaction is well established (Robert‐Seilaniantz *et al*. 2011) with increased in SA favouring necrotrophic interactions (El Oirdi *et al*. 2011). This would imply that SA could also favour disease progression in wheat challenged with the necrotroph *Ptr* and SA effects were prominent only with Her. This hypothesis was supported by the effects seen with exogenous application of SA which augmented some features of disease development. However, with exception of lower levels of 13‐lipoxygenase expression, these SA related changes could not be linked to reduced JA‐linked expression with other JA biosynthetic intermediates (AOC, 13(S)‐HPOT) and signalling genes (*e.g*., JAZ) induced in Her. Thus, the initiation of disease is undoubtedly more complex with multiple factors playing different roles. For example, it may be that elevation of SA signalling along with the suppression of IAA events act together to increase susceptibility as has been suggested in other pathosystems (Llorente *et al*. 2008; Rahman *et al*. 2012).

Taken together, we have characterised resistance and susceptibility to *Ptr* infection in wheat seedlings by following complementary approaches: phenotypic assessments of highly diverse lines, and subsequent RNA‐seq and metabolomics analyses. The derived model could be used to inform the development of new TS resistant cultivars by suggesting a focus on certain aspects of defence, as well as guiding further work on responses to *Ptr*.

## AUTHOR CONTRIBUTIONS

LCF, FMS, SMMS and LAJM designed the study. LCF and MB performed research. LCF, MB, and LAJM analysed data. LCF and LAJM wrote the paper.

## FUNDING INORMATION

This work was supported by an AberDoc PhD scholarship to LCF, a Joy Welch award to LCF and LAJM and an Embrapa project fund (SEG 03.16.05.009.00.00) to FMS.

## CONFLICT OF INTEREST STATEMENT

The authors declare no conflict of interest.

## Supporting information


**Figure S1.** Percentage of responses observed in the wheat lines A: Hereward (Her) and B: Robigus (Rob) at 24 and 72 h post inoculation with *Pyrenophora tritici‐repentis*, using fluorescent microscopy.

**Figure S2.** Expression of genes associated with salicylic acid in the wheat line Hereward (Her) at 48 and 96 h post inoculation with *Pyrenophora tritici‐repentis* (I) or mocks (M).

**Figure S3.** Lignin assessments of Hereward (Her) and Robigus (Rob) seedlings challenged with *Pyrenophora tritici‐repentis* (*Ptr*). A: Relative fractions of alcohol insoluble residue (AIR) and acetyl bromide soluble lignin (ABSL) derived from total biomass collected from each genotype that were mock‐inoculated (M) or Ptr‐inoculated (I), at 0 and 96 h post inoculation. Data represent mean and standard deviation of biological and technical replicates. Treatments labelled with same letters denote statistically non‐significant difference between mean values of each trait. Micrographs of Wiesner stained leaves infected with *P. tritici‐repentis* from the wheat lines B: Her C: Rob. Scale bar = 100 μM.

**Table S1.** RNA sequencing quality report and metadata.

**Table S2.** Functional annotation of differentially expressed genes (DEGs) between the wheat lines Robigus and Hereward before inoculation (Rob/Her_0) and between the controls from each line in comparison to the samples collected at 48 and 96 h post inoculation.

**Table S3.** Unique bins of differentially expressed genes (DEGs) found in the wheat lines Robigus and Hereward and their MapMan annotation.

**Table S4.** Statistical analysis and functional annotation of differentially accumulated metabolites (DAMs) found between the wheat lines Robigus (Rob) and Hereward (Her) before inoculation (Rob/Her_0) and between the controls from each line in comparison to the samples collected at 24, 48, 72 and 96 h post inoculation.

**Table S5.** Mass‐to‐charge (*m/z*) analytes generated by Flow Infusion Electrospray Ionisation High‐Resolution Mass Spectrometry (FIE‐HRMS) of leaves sampled from the wheat lines Robigus (Rob) and Hereward (Her) at 0, 24, 48, 72 and 96 h post inoculation with *Pyrenophora tritici‐repentis* (I) or mock‐inoculated (M).

## Data Availability

The RNA‐seq data that support the findings of this study are openly available in NCBI Gene Expression Omnibus at www.ncbi.nlm.nih.gov/bioprojet, reference number PRJNA836737. All other data that support the findings of this study are available from the corresponding author upon reasonable request.
