# Supplementary figures and images for: Omic characterisation of multi‐component defences against the necrotrophic pathogen Pyrenophora tritici‐repentis in wheat
Source: Plant Biol (Stuttg). 2025 Feb 7;27(3):347–61. doi: 10.1111/plb.13746 (PMC11950905; doi:10.1111/plb.13746)

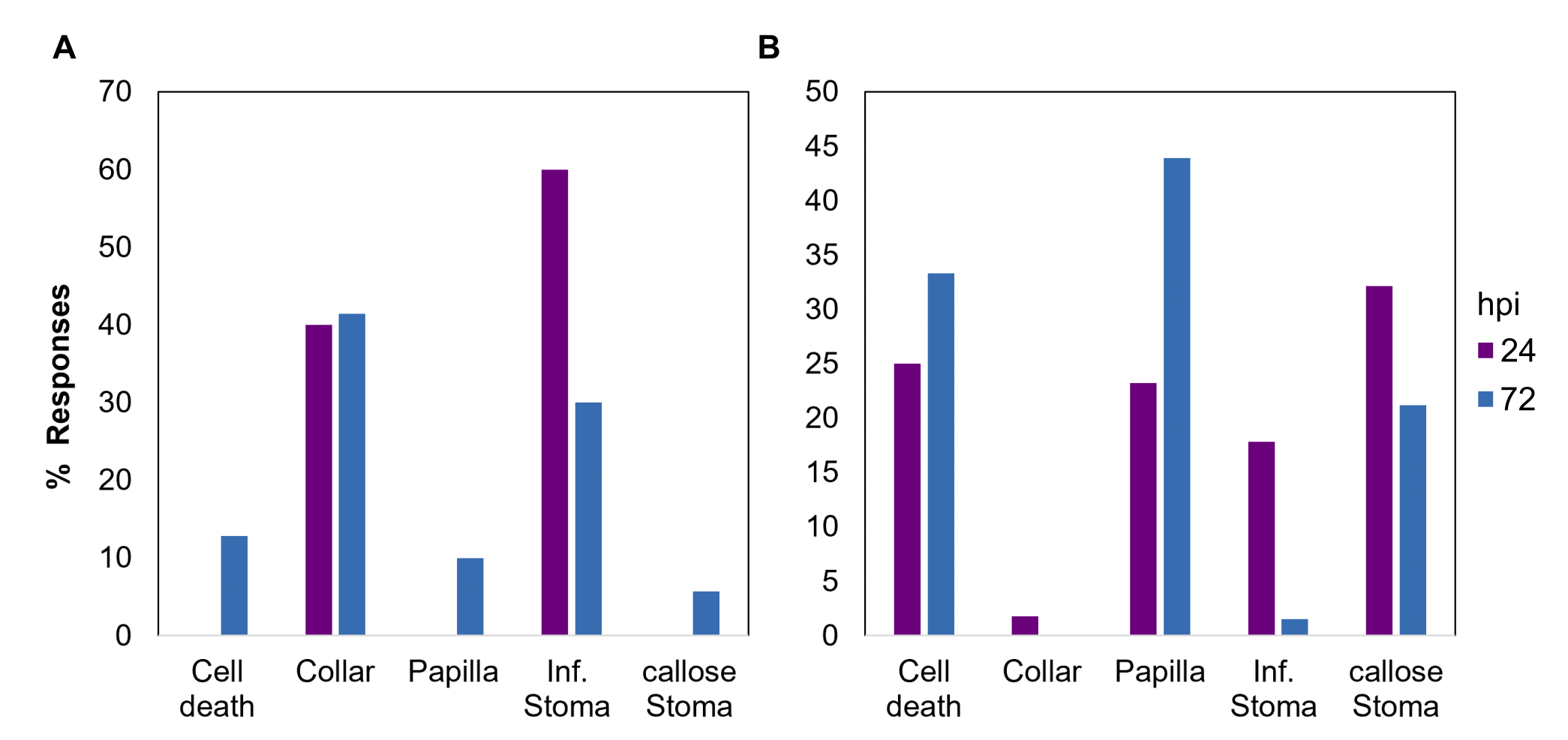

Supplement: Supplementary file 1 — Figure S1. Percentage of responses observed in the wheat lines A: Hereward (Her) and B: Robigus (Rob) at 24 and 72 h post inoculation with Pyrenophora tritici‐repentis, using fluorescent microscopy. [file PLB-27-347-s002.tiff]

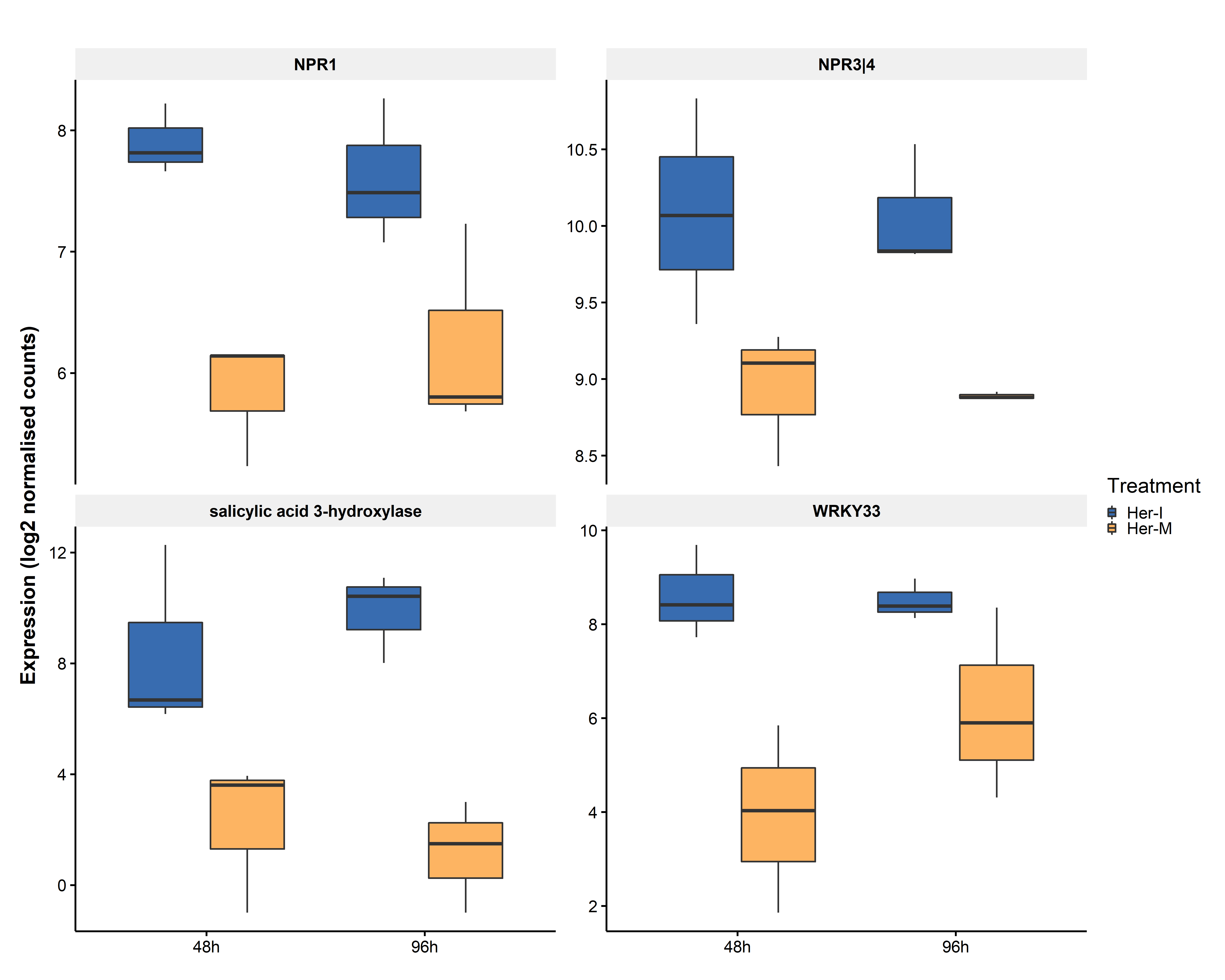

Supplement: Supplementary file 2 — Figure S2. Expression of genes associated with salicylic acid in the wheat line Hereward (Her) at 48 and 96 h post inoculation with Pyrenophora tritici‐repentis (I) or mocks (M). [file PLB-27-347-s006.png]

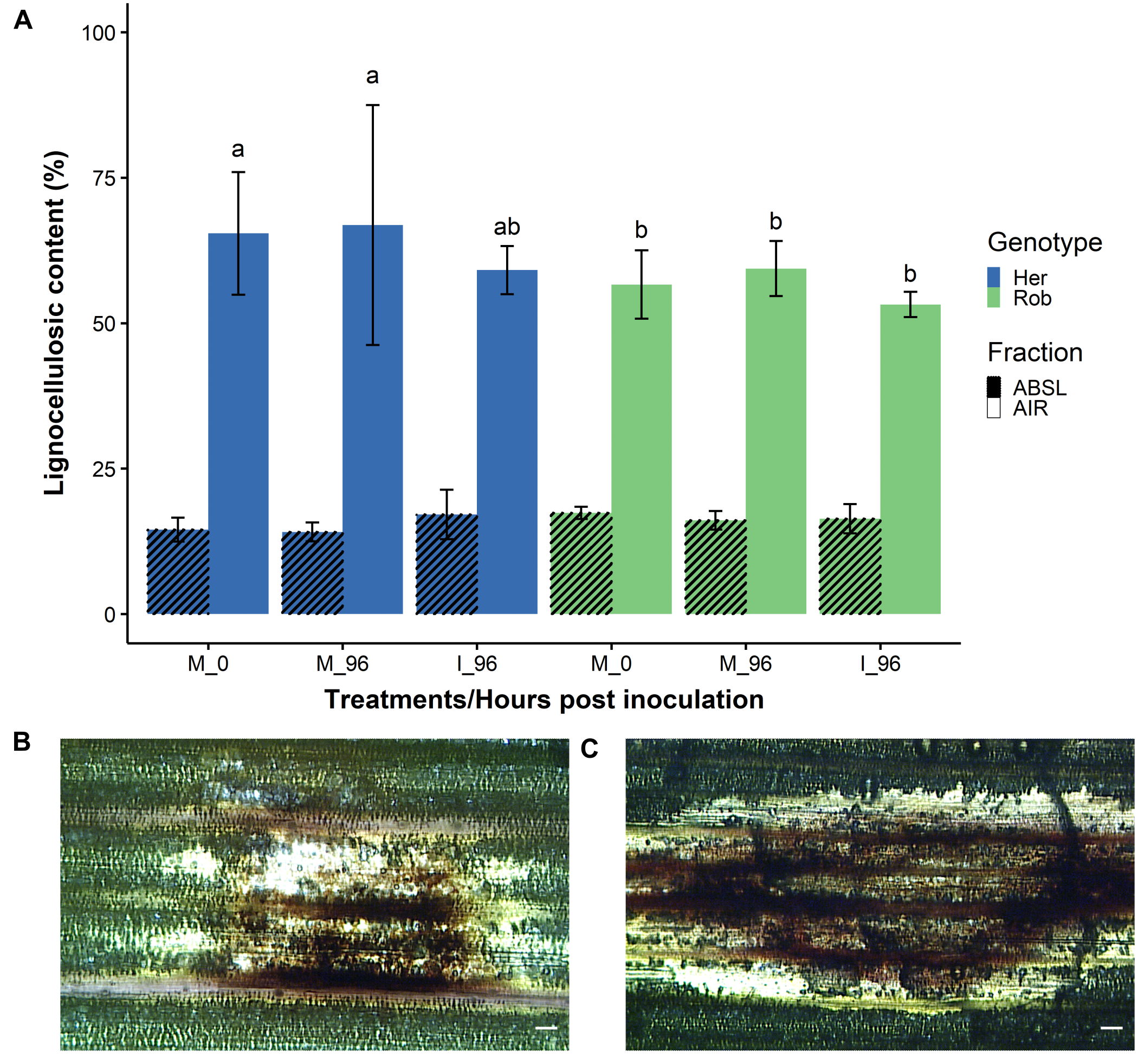

Supplement: Supplementary file 3 — Figure S3. Lignin assessments of Hereward (Her) and Robigus (Rob) seedlings challenged with Pyrenophora tritici‐repentis (Ptr). A: Relative fractions of alcohol insoluble residue (AIR) and acetyl bromide soluble lignin (ABSL) derived from total biomass collected from each genotype that were mock‐inoculated (M) or Ptr‐inoculated (I), at 0 and 96 h post inoculation. Data represent mean and standard deviation of biological and technical replicates. Treatments labelled with same letters denote statistically non‐significant difference between mean values of each trait. Micrographs of Wiesner stained leaves infected with P. tritici‐repentis from the wheat lines B: Her C: Rob. Scale bar = 100 μM. [file PLB-27-347-s008.tiff]
